# A Robust, Tough and Multifunctional Polyurethane/Tannic Acid Hydrogel Fabricated by Physical-Chemical Dual Crosslinking

**DOI:** 10.3390/polym12010239

**Published:** 2020-01-19

**Authors:** Jie Wen, Xiaopeng Zhang, Mingwang Pan, Jinfeng Yuan, Zhanyu Jia, Lei Zhu

**Affiliations:** 1 Institute of Polymer Science and Engineering, School of Chemical Engineering and Technology, Hebei University of Technology, Tianjin 300130, China; wenjie0329@126.com (J.W.); xpzhangup@163.com (X.Z.); yuanjf@hebut.edu.cn (J.Y.); 15122410536@163.com (Z.J.); 2 Hebei Key Laboratory of Functional Polymers, Hebei University of Technology, Tianjin 300130, China; 3 Department of Macromolecular Science and Engineering, Case Western Reserve University, Cleveland, OH 44106-7202, USA; lxz121@case.edu

**Keywords:** tannic acid, double crosslinking, polyurethane hydrogel, mechanical properties, multifunction

## Abstract

Commonly synthetic polyethylene glycol polyurethane (PEG–PU) hydrogels possess poor mechanical properties, such as robustness and toughness, which limits their load-bearing application. Hence, it remains a challenge to prepare PEG–PU hydrogels with excellent mechanical properties. Herein, a novel double-crosslinked (DC) PEG–PU hydrogel was fabricated by combining chemical with physical crosslinking, where trimethylolpropane (TMP) was used as the first chemical crosslinker and polyphenol compound tannic acid (TA) was introduced into the single crosslinked PU network by simple immersion process. The second physical crosslinking was formed by numerous hydrogen bonds between urethane groups of PU and phenol hydroxyl groups in TA, which can endow PEG–PU hydrogel with good mechanical properties, self-recovery and a self-healing capability. The research results indicated that as little as a 30 mg·mL^−1^ TA solution enhanced the tensile strength and fracture energy of PEG–PU hydrogel from 0.27 to 2.2 MPa, 2.0 to 9.6 KJ·m^−2^, respectively. Moreover, the DC PEG–PU hydrogel possessed good adhesiveness to diverse substrates because of TA abundant catechol groups. This work shows a simple and versatile method to prepare a multifunctional DC single network PEG–PU hydrogel with excellent mechanical properties, and is expected to facilitate developments in the biomedical field.

## 1. Introduction

Hydrogels can absorb a large amount of water molecules but not be dissolved in water, because they possess hydrophilic segments in their 3D crosslinked network [1,2]. On account of their admirable swelling property and eco-friendliness, hydrogels are applied in many fields such as medicine [3,4], agriculture [5,6] and cosmetics [7], etc. However, it is still a big challenge to prepare hydrogels for load-bearing applications including sensors [8,9], structural biomaterials [10,11], and soft robotics [12,13,14], because commonly synthetic hydrogels possess low mechanical robustness, poor stretchability and toughness, which are derived from their inherent structural heterogeneity or absence of effective energy dissipation mechanism, restricting their scopes of practical application [15]. Recently, many works have been done to exploit novel polymerization approaches for preparing hydrogels with preferable mechanical performance, for instance, nanocomposite hydrogel [16], double network (DN) hydrogel [17], double-crosslinked (DC) hydrogel [18], etc.

Among these diversified hydrogels, polyethylene glycol polyurethane (PEG–PU) hydrogel, a unique block copolymer hydrogel containing the urethane group, is one of the most researched and widely applied hydrogels [19]. Due to its adjustable chemical structure, good biocompatibility and low toxicity [20,21,22], PEG–PU hydrogel is commonly used as biomaterials such as drug delivery devices [23,24,25,26,27,28], neuroprosthetic devices [29], tissue engineering scaffolds [30], etc. As for tissue engineering scaffolds, including muscle, cartilage, and tendon, outstanding mechanical properties are essential when fabricating a PEG–PU hydrogel to repair the tissue. Unfortunately, ordinarily single crosslinked PEG–PU hydrogels possess relatively poor mechanical properties, which limits their application in many fields [31,32]. Hence, some new DC PEG–PU hydrogels have been prepared to improve their mechanical properties. Li et al. used 3,3′-disulfanediyldipropane-1,2-diol (DSO) as the second crosslinker for preparation of DC PEG–PU hydrogels, the highest value of tensile strength among these hydrogels was 0.25 MPa [33]. Divakaran et al. also prepared a DC PEG–PU hydrogel with the tensile strength of 0.7 MPa and the breaking elongation of 284% at equilibrium swelling state, in which 1,2,6-hexanetriol and curcumin were used as crosslinkers [34]. These studies suggest that the DC PEG–PU hydrogels are able to be fabricated by using the polyhydroxy compound as the second crosslinking agent and the mechanical strength of obtained PEG–PU hydrogels is higher than the single crosslinked PEG–PU hydrogels, but their strength, stretchability and toughness need to be further improved.

Based on the above research, we proposed a novel approach for preparation of DC PEG–PU hydrogel with excellent mechanical properties, high adhesive strength, good self-recovery and a self-healing ability. On account of such considerations, we deliberately chose a natural polyphenolic compound tannic acid (TA) as the second crosslinker, except for utilizing usual crosslinker trimethylolpropane (TMP). TA derives from bark and fruit of a variety of trees, and is easily soluble in water, cheap, and low-toxicity [35]. In terms of chemical structure, TA contains abundant catechol and pyrogallol groups, which can bond with urethane groups of PEG–PU hydrogel to form physical hydrogen bonds and endow PEG–PU hydrogel with good adhesiveness [36]. Further, dynamic characteristic of hydrogen bonds can impart self-recovery and self-healing capability to PEG–PU hydrogel. Consequently, we fabricated novel DC PEG–PU hydrogels with different content TA. The first crosslinker TMP was introduced into a single network by chemical crosslinking. Second, the second crosslinking was formed by the facile immersion method of adding TA into the crosslinked single network system, which led to generation of DC PU/TA hydrogels. Furthermore, the increase of TA content in PEG–PU hydrogel can improve their crosslinked density and mechanical properties. To the best of our knowledge, it is barely reported that using TA as the second crosslinker through the physical crosslinking method can be used to fabricate DC single network PEG–PU hydrogel with homogeneous structure and versatility. Moreover, the obtained PEG–PU hydrogel possessed higher robustness and toughness, better self-recovery, self-healing capability and adhesiveness than some DC and DN PEG–PU hydrogels [33,34,37]. This practical and universal strategy is anticipated to unfold a novel route for developing multifunctional PEG–PU hydrogels with dramatically enhanced mechanical properties, promoting their development in load-bearing applications.

## 2. Materials and Methods

### 2.1. Materials

Polyethylene glycol (PEG, M_n_ = 4000, Solarbio, Beijing, China)), isophorone diisocyanate (IPDI, 99%, Aladdin, Shanghai, China), dibutyltin dilaurate (DBTDL, 96%, Tianjin Guangfu Fine Chemical Research Institute, Tianjin, China), trimethylolpropane (TMP, 98%, Tokyo Chemical Industry, Tokyo, Japan), *N*,*N*-dimethylformamide (DMF, GC, 99.9%, Aladdin, Shanghai, China), tannic acid (TA, AR, Aladdin, Shanghai, China). PEG was dehydrated in vacuum oven at 120 °C for 2 h before using. DMF was dried with 4 Å molecular sieve, then distilled under reduced pressure and was preserved with a 4 Å molecular sieve before use. The other chemical reagents were used as we received.

### 2.2. Fabrication of Polyethylene Polyurethane (PEG–PU) Hydrogels

The following fabrication was conducted under N_2_ atmosphere, and the molar ratio of NCO and OH was 1.15. The dehydrated PEG4000 (5 g) was dissolved in dried DMF (15 mL) at 80 °C with continuous stirring to form a solution in a four-necked flask. After dropwise addition of IPDI (1.3 g) and 8 µL catalyst DBTDL, the reaction mixture was mechanically stirred using a powerful mechanical anchor stirrer with two stirring blades (JB90-SH, Shanghai Biaomo Instrument Co., Ltd., Shanghai, China) for 2 h at 80 °C. TMP (0.34 g) was dissolved in DMF (5 mL) and dropwise added to the reaction mixture at 60 °C with stirring for 30 min. The mixture was poured into glass Petri dishes, which were put on the experimental platform. After 12 h of standing at room temperature, the reaction mixture was cured to form gel. The gel was dried in vacuum drying oven (DZG-403 B, Tianjin Tianyu Laboratory Instrument Co., Ltd., Tianjin, China) to remove DMF at 80 °C. The dry gels were immersed in excessive deionized water for 72 h with continual replacement of fresh deionized water to obtain the PEG–PU hydrogels.

### 2.3. Fabrication of TA–PU Hydrogels

The as-prepared PEG–PU hydrogels were quickly frozen under liquid nitrogen and dried by a lyophilizer. Subsequently, the obtained freeze-dried gels (1 g) were placed into TA aqueous solutions (300 mL) with different concentrations (1, 4, 7, 10, 20, 30 mg·mL^−1^) at room temperature for 18 h. The obtained hydrogels were referred to as TAx–PU, where x is the concentration of TA solution. For instance, TA4-PU means the PEG–PU gel immersed into a 4 mg·mL^−1^ of TA solution. After that, the TAx–PU hydrogels were soaked in distilled water (300 mL) at room temperature for 12 h to achieve swelling equilibrium. These swelling equilibrium hydrogels are denoted as TAx–PU(S).

### 2.4. Characterizations and Measurements

#### 2.4.1. Fourier Transform Infrared Spectroscopy (FTIR)

The chemical structure of the TAx–PU(S) dry gels was analyzed by FTIR (Bruker, Karlsruhe, Germany). FTIR spectra of the samples were recorded using a Bruker Tensor-27 spectrometer at room temperature in the range from 4000 to 400 cm^−1^ [38]. The samples were milled with desiccative potassium bromide (KBr) power and pressed into pellets using a tabulating machine.

#### 2.4.2. Scanning Electron Microscope (SEM)

SEM was employed to observe the morphology of the samples and was conducted on a Nano 450 FE-SEM instrument (FEI, Hillsboro, OR, USA) operating at 10 kV. For the SEM observation, the samples were sputter-coated with Au after being freeze-dried in a lyophilizer (FD-1A-50, Beijing Boyikang Laboratory Instrument Co., Ltd., Beijing, China) at −40 °C.

#### 2.4.3. X-ray Diffraction Pattern (XRD)

The crystallinity of PU and TA–PU dry gels was determined using X-ray diffraction technique (Brucker, Karlsruhe, Germany). The determination was carried out at 25 °C on a Bruker D8 Focus X-ray diffractometer operated at 40 mA and 40 kV with Cu Kα radiation (wavelength, λ = 1.5418 Å). The samples were scanned among 2θ = 3–40° at a scanning speed of 12° min^−1^.

#### 2.4.4. Swelling Behavior

The swelling behavior of PU and TA–PU gels was investigated by water and TA contents which were obtained by a gravimetric process. The calculation formula for water content and TA content in the samples can be seen in the Supporting Information.

#### 2.4.5. Mechanical Performance

The mechanical performance of the samples was measured using a microcomputer control universal testing machine (XinSanSi CMT6104, Shenzhen, China) at 25 °C (humidity 45%). For tensile tests, tensile stress–strain curves were acquired under such conditions: hydrogel size, 50 mm × 10 mm × 1 mm; stretching speed, 100 mm·min^−1^; gauge length, 20 mm. The elastic modulus E was obtained via calculating the slope over 5–10% of strain ratio for the stress–strain curves. For the loading–unloading tests, the test condition was the same as tensile test. The dissipated energy was acquired by calculating the area of the loading–unloading profile. As for tearing tests, the samples were cut into a trouser shape on the basis of GBT-529-2008A standard, and the stretching speed was also 100 mm·min^−1^. The fracture energy (Γ) was obtained using the formula Γ = F_ave_/d, where F_ave_ is the average loading force obtained by tearing tests and d is thickness of the samples [39]. To impede water evaporation during tests, a spot of silicone oil was smeared on the samples, and each test was duplicated at least five times.

#### 2.4.6. Dynamic Mechanical Analysis (DMA)

Dynamic mechanical analysis (DMA) of the samples was conducted on a dynamic mechanical analyzer (DMA2000B, Tritec Instruments, York, UK) at an oscillation frequency of 1 Hz. The rectangular samples (40 mm × 7 mm × 2 mm) were cooled to −100 °C under liquid nitrogen firstly, followed by heating up to 110 °C at the rate of 3 K·min^−1^.

#### 2.4.7. Adhesiveness Test

Adhesiveness of TA–PU hydrogels was examined by a series of tensile adhesion tests according to the mode as shown in Figure S5 which were carried out through using a microcomputer control universal testing machine (XinSanSi CMT6104, Shenzhen, China) in accordance with ASTM-C6SS standard. During the tests, the substrates were commercially available tinplate (SPTE), titanium (Ti), glass, polymethyl methacrylate (PMMA), polycarbonate (PC) and porcine skin tissue. These engineering solid samples (100 mm × 25 mm × 1 mm) were cleaned using distilled water and anhydrous alcohol, respectively, followed drying before the test. Additionally, the tested porcine skin samples (25 mm × 25 mm) were adhered to aluminum fixing devices with cyanoacrylate glue before measurement [36]. To attach these substrates, the hydrogels (20 mm × 20 mm × 1 mm) were put between two substrate sheets and compressed using a 200 g weight for 5 min. The attached substrates were fastened with fixture, followed by being separated at a crosshead rate of 5 mm min^−1^. The adhesion strength was acquired by using the determined maximum loading force divided by the hydrogel area. Each sample was measured in parallel five times.

## 3. Results and Discussion

### 3.1. Fabrication of TA–PU Hydrogels

**Scheme 1:**
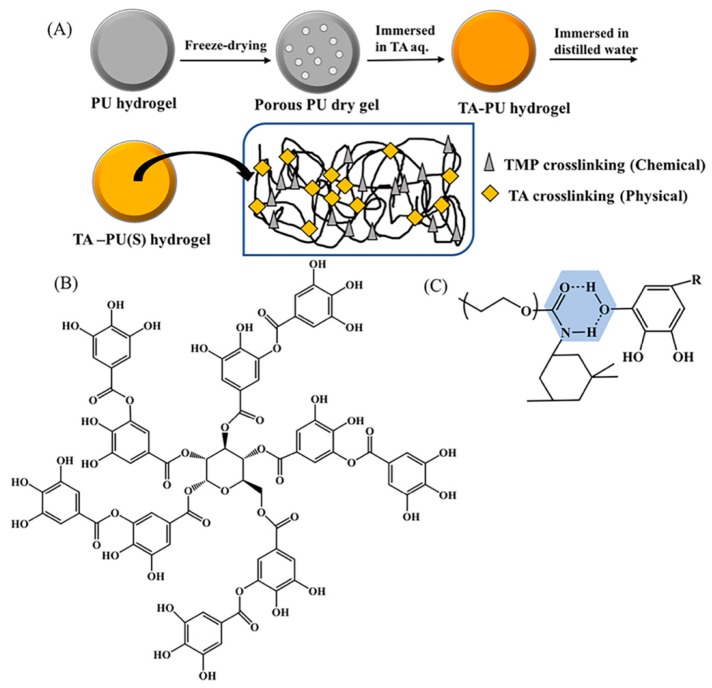
(**A**) Fabrication procedure of TA–PU hydrogels; (**B**) chemical structure of TA; (**C**) the hydrogen bonds between PU and TA.

The preparation procedure of TA–PU hydrogel is systematically shown in Scheme 1A. First, PEG–PU hydrogel with covalent crosslinking single network was synthesized by a simple pre-polymerization, in which TMP was used as the first crosslinker. Second, the chemically crosslinked PU hydrogel became porous PU dry gels through a fast-freezing treatment and then lyophilized process. The formation of porous structure arose because the frozen water molecules (ice crystals) in PU hydrogel were removed by sublimating in vacuo, and then the pores were left where ice crystals were first occupied. The acquired porous PU dry gels were soaked in TA solutions with different concentrations for 18 h (Figure S1) to create the second physical crosslinking which was formed by intermolecular hydrogen bonding between PU and TA. In this research, TA was chosen as the second crosslinker by means of abundant phenolic hydroxyl groups in its chemical structure (Scheme 1B), which can generate a large amount of hydrogen bonds with the urethane groups in PU (Scheme 1C). Finally, the obtained hydrogels were immersed in distilled water for 12 h (Figure S2) to remove the unreacted TA molecules and improve the rearrangement of hydrogen bonding in TAx–PU hydrogels as well, generating equilibrium swollen double-crosslinked TAx–PU(S) hydrogels.

### 3.2. Structural Characterization

**Figure 1:**
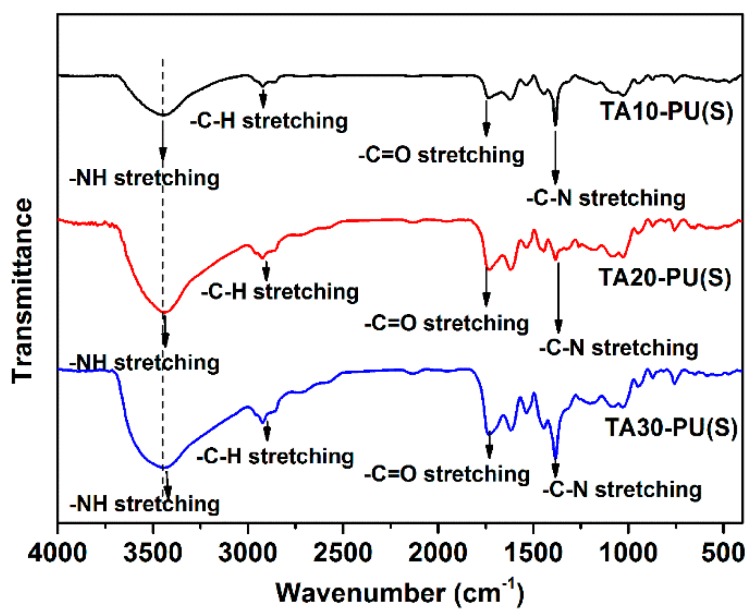
FTIR spectra of TA10–PU(S), TA20–PU(S), and TA30–PU(S) dry gels.

FT-IR spectroscopy was used to verify chemical structure of polymer matrix. Moreover, evidence of hydrogen bonds can be collected by following the variation in intensity and frequency of the absorption peaks in the mid-IR spectral area [40,41]. The FT-IR spectra of TA10–PU(S), TA20–PU(S) and TA30–PU(S) dry gels are shown in Figure 1. The absorption peak at 3420 cm^−1^ was attributed to –N–H stretching vibration. Moreover, the –C=O stretching vibration in urethane (–NHCOO–) group was observed at 1730 cm^−1^. The –C–N stretching vibration happened near 1230 cm^−1^. These observations demonstrated that the urethane group was generated successfully by the reaction between hydroxyl (–OH) group of PEG and isocyanate (–NCO) group in IPDI. Notably, a slight blue shift of infrared band was witnessed in –NH stretching vibration region with increase of TA solution concentration (x), which occurred because of the generation of hydrogen bonds between phenol hydroxyl groups in TA and urethane (–NHCOO) groups from PU, and the number of hydrogen bonds in TAx–PU(S) gels increased with enhancement of TA content. The result clearly proved that TA existed in the double-crosslinked TAx–PU(S) gels.

### 3.3. Microstructure Characterization

X-ray powder diffractions (XRD) of PU and TAx–PU(S) dry gels are shown in Figure S3, illustrating the influence of TA content on the microstructure. In the case of pure PU dry gel, there were two characteristic diffraction peaks. They focused on 2θ = 19.2° and 23.3°, which belonged to 120 and 132 crystal planes reflections of PEG monoclinic cells respectively [42]. However, the TA10–PU(S) dry gel had only a distinct and broad characteristic diffraction peak near 2θ = 21°, while the characteristic diffraction peaks for TA20-PU(S) dry gel became less obvious. This indicated that the crystallinity of TAx–PU(S) gels reduced with the increase of TA content (namly the second crosslinking degree). Furthermore, the XRD spectra of TAx–PU(S) gels possessed a general left shift compared to PU hydrogel. This change is caused by this fact that TA as a hard segment existed in polyurethane chain, which led to the increase in steric hindrance of soft segment crystallization and the reduction of crystallization of TAx–PU(S) gels.

### 3.4. Swelling Behavior

**Figure 2:**
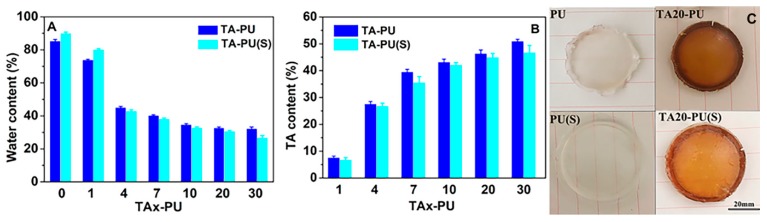
Water content (**A**) and tannic acid (TA) content (**B**) of TAx–PU hydrogels; photos of PU and TAx–PU gels before and after swelling in distilled water (**C**).

Swelling behaviors of hydrogels affect their mechanical performance, so it is extremely important to research their swelling behaviors before optimizing their mechanical properties [43]. Here, the influences of water and TA content in PU and DC TA–PU hydrogels on their swelling behavior were investigated. As shown in Figure 2, when the TA solution concentrations were enhanced from 0 to 30 mg·mL^−1^, TA content in the hydrogels continually increased (Figure 2B), whereas their water content gradually reduced (Figure 2A), whether these gels were in as-prepared or equilibrium swelling states. These observations indicated that the swelling behavior of the hydrogels were profoundly influenced by the second physical crosslinking building on soaking in TA solutions. The reason was that PU was more inclined to generate hydrogen bonding with dendritic polyphenol compound TA than water molecules. Consequently, TA molecules occupied more and more hydrogen bond receptors in the PU chains upon the increase in concentration of TA solution in the course of immersion.

The strong interactions between PU and TA imparted excellent resistance of the TA–PU hydrogels against water swelling. The as-prepared PU hydrogels were soaked in distilled water for 72 h to arrive the swelling equilibrium state. It can be observed from Figure 2C that the volume and color of TA–PU(S) hydrogel had slightly been changed, which suggested that the swelling of TA–PU(S) hydrogels was restrained to some extent and some unbonded TA molecules was released when these hydrogels were at swelling equilibrium state. It is worth mentioning that the water contents of TA4–PU(S), TA7–PU(S), TA10–PU(S), TA20–PU(S), TA30–PU(S) hydrogels lowered than their as-prepared states (Figure 2A). This was likely caused by the formation of tighter cross-linking coagulation in the TAx–PU(S) hydrogels with the increase of TA content, which prevented entrance of water molecules [44]. Owing to the change of water content and the release of TA molecules, the TA content in TAx–PU(S) hydrogels slightly decreased compared with TAx–PU hydrogels (Figure 2B). Additionally, it was also observed from Figure S4 that the swelling ratio of TAx–PU(S) hydrogels declined with the increase in TA content, and the swelling ratio of TAx–PU(S) hydrogels was dramatically lower than that of pure PU(S) hydrogels. This indicated that the density of physical crosslinking in TAx–PU(S) hydrogels remarkably increased with the increase of TA content.

**Figure 3:**
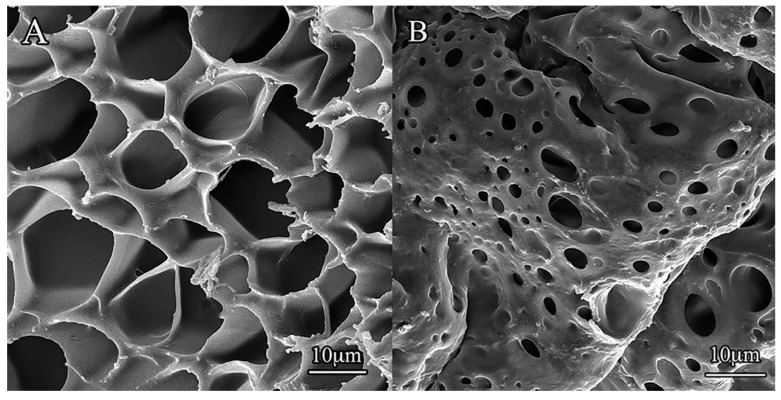
SEM photographs of lyophilized PU (**A**) and TA10-PU(S) (**B**) hydrogels.

To further understand swelling character of TA–PU hydrogels, morphology of freeze-dried PU and TA10–PU(S) hydrogels were studied by SEM. It can be seen from Figure 3 that the pure PU hydrogel owned loose porous network and the pore size was within 7–20 µm (Figure 3A), while the pore size (1–7 µm) became small and the pore wall appeared to be compact for TA10–PU(S) hydrogel (Figure 3B). The variety in morphology was due to the increase of crosslinking degree after introducing TA. When porous PU gels were soaked into TA solution, the porous structure effectively provided a sufficient and rapid contact between PU and TA molecules, which avoided heterogeneity caused by the surface layer of PU/TA gels. Therefore, the preparation of porous PU gel was crucial to achieving strong DC PU hydrogels.

### 3.5. Mechanical Properties

**Figure 4:**
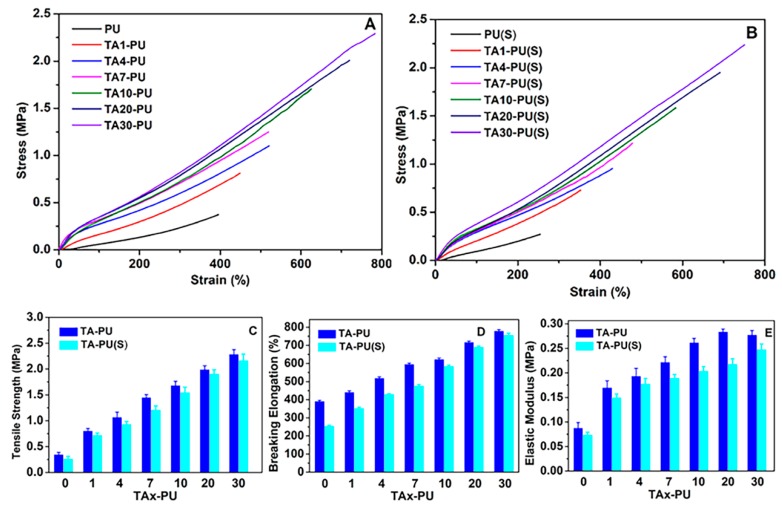
Tensile strain–stress curves of TA–PU hydrogels at as-prepared (**A**) and swelling equilibrium (**B**) states. Mechanical properties of pure PU and TA–PU hydrogels: (**C**) tensile strength; (**S**) breaking strain; (**E**) elastic modulus.

TA content has a significant effect on mechanical performance of DC PUHs due to polyphenolic hydroxyl groups existing in the chemical structure of TA. Hence, we examined the influence of TA content on mechanical properties through stretching, tearing, and cyclic loading–unloading ways. Figure 4A,B exhibits the tensile stress–strain curves of pure PU and TA–PU hydrogels at as-prepared and swelling equilibrium states. It can be observed that introducing TA into PU hydrogels led to prominent enhancement in the mechanical performance of TA–PU compared with the pure PU hydrogel, and their mechanical performance gradually rose with increase in the concentration of TA solution. This provides an opportunity for adjusting the robustness and toughness of TA–PU hydrogels by suitable swelling behavior (see Figure 2). When the concentration of TA solution was 30 mg·mL^−1^, the tensile strength of the as-prepared TA–PU hydrogel increased up to 2.3 MPa following the breakage elongation of 783% and the elastic modulus of 0.27 MPa, which were the highest values among the as-prepared TA–PU hydrogels. Remarkably, the tensile strength of the equilibrium swollen TA30-PU hydrogel can reach 2.2 MPa with breakage elongation of 750% and the elastic modulus of 0.24 MPa, which is barely lowered compared with its as-prepared state. This fact suggested that the TA–PU hydrogels containing a proper TA content can possess excellent mechanical properties even if they are at swelling equilibrium state, which could be because numbers of hydrogen bonding between PU and TA were enhanced with the increase in TA content. Moreover, the tensile strength, elongation at break and elastic modulus of TA–PU hydrogels at as-prepared and swelling equilibrium states are summarized in Figure 4C–E. These properties were improved significantly with the increase in TA content whether the hydrogels were at as-prepared or swelling equilibrium state, which was also attributed to formation of more hydrogen bonds and increase of crosslinking density. This regular change indicated the good homogeneity in the network structure of TA–PU hydrogels. Obviously, the mechanical strength, breaking strain and elastic modulus of equilibrium swollen TAx–PU hydrogels slightly decreased compared to the as-prepared TAx–PU hydrogels. This decrease is because the reduction of TA content in TAx–PU(S) hydrogels led to the decline of the second crosslinking density. Nevertheless, it was reported that DC PU-curcumin hydrogels exhibited a complex change in the mechanical performance with increase of curcumin content, which was because of inhomogeneity in the network structure of those hydrogels [34].

**Figure 5:**
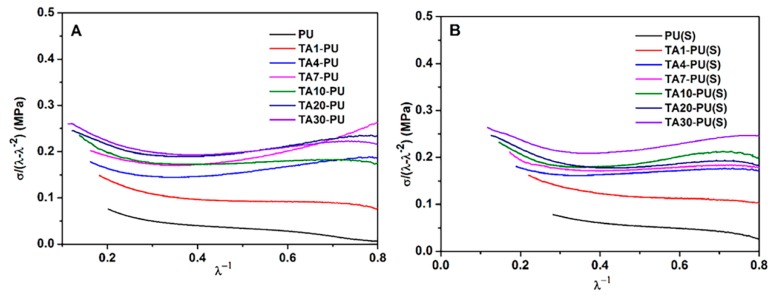
Mooney–Rivlin curves of pure PU and TA–PU hydrogels at as-prepared (**A**) and swelling equilibrium (**B**) states.

In order to preferably comprehend the deformation mechanism of DC TA–PU hydrogels, the stress–strain curves were further studied through using phenomenological Mooney–Rivlin equation:

(1) $$\sigma_{\mathrm{red}}=\frac{\sigma }{\lambda -\lambda^{-2}}={2C}_{1}+{2C}_{2}\frac{1}{\lambda }$$

where σ_red_ is the reduced stress, σ is the stress, *λ* is the extension ratio, the relationship between *λ* and the strain ε is *λ* = ε + 1, and C_1_ and C_2_ are material constants. The quantity 2C_1_ is equal to the shear modulus (=E/3), and C_2_ is related to the strain softening (C_2_ > 0) or hardening (C_2_ < 0) beyond the Gaussian elasticity region [45,46]. Plots of σ_red_ against *λ*^−1^ for pure PU and TA–PU hydrogels at as-prepared and swelling equilibrium states are shown in Figure 5. In the case of the pure PU and TA1-PU hydrogels, they exhibited obvious strain hardening during the deformation. This occurrence may be induced by the fact that the crystalline domains still existed in the hydrogels after the slight degree of physical crosslinking, the polyurethane chains between crystalline domains were pre-stretched and orientated to supply further deformation and enhancement. When large deformation happened, the crystalline slippage or/and chain breakage introduced crack propagation, leading to the rapid fracture of the as-prepared and equilibrium swollen PU and TA1–PU gels. For TA4–PU, TA7–PU, TA10–PU, TA20–PU and TA30–PU at as-prepared and swelling equilibrium states, at small deformation (*λ*^−1^ > 0.4), strain softening was observed, which may be because large amount of interchain hydrogen bonds were broken during the tensile deformation. At large deformation (*λ*^−1^ < 0.4), the TA–PU hydrogels exhibited obvious strain hardening due to the finite extensibility and then orientation enhancement of polyurethane chains, similar to the pure PU and TA1-PU hydrogels [18]. At the same time, the rearranged PU chains were associated with TA to form the hydrogen bonds again. These results illustrated that the swelling treatment had little effect on deformation of these hydrogels.

### 3.6. Toughness of TA–PU Hydrogels

**Figure 6:**
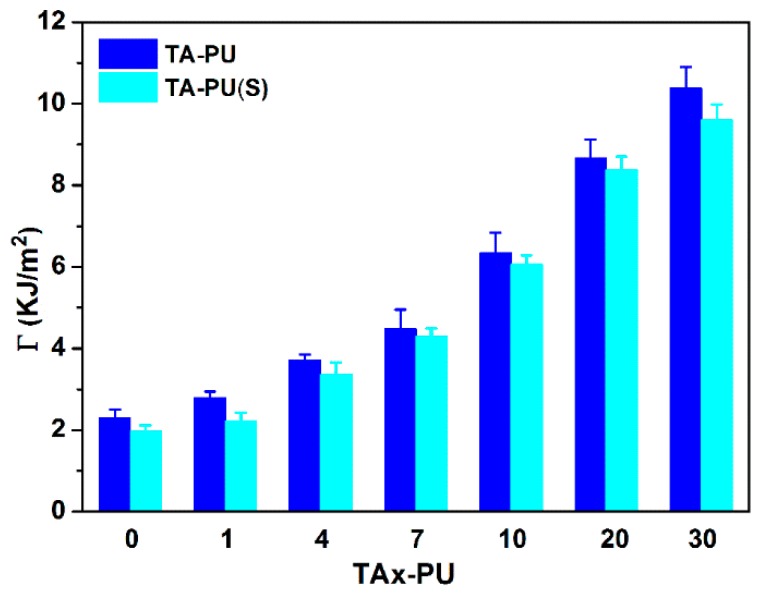
Fracture energy of TAx–PU hydrogels at as-prepared and equilibrium swelling states.

The introduction of TA not only improved the mechanical strength of hydrogels, but also had a distinct influence on their toughness because of the existence of massive hydrogen bonds in the TA–PU hydrogels. Here, the toughness of TA–PU hydrogels was investigated by fracture energy. As shown in Figure 6, the fracture energy of these hydrogels was enhanced with increase in the concentration of TA solution. The result indicated that both as-prepared and equilibrium swollen states of TA–PU hydrogels showed excellent toughness when the second crosslinking density increased. In terms of TA30–PU hydrogel, its fracture energy (Γ) was nearly 10.5 KJ·m^−2^, and it still possessed a relatively high fracture energy (9.6 KJ·m^−2^) when it was at equilibrium swelling state, which was about five times that of pure PU hydrogel.

**Figure 7:**
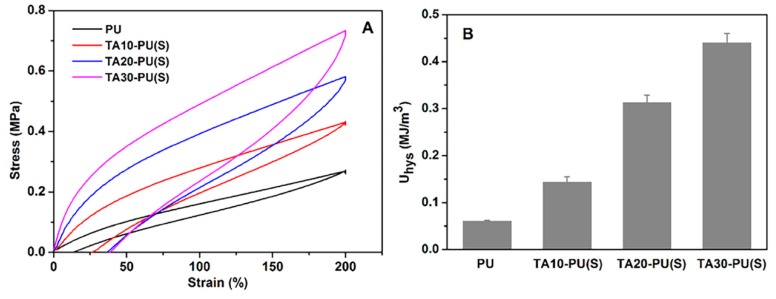
Stress–strain curves from loading–unloading tests (**A**) and calculated dissipated energy (**B**) of pure PU and TAx–PU(S) hydrogels under 200% of strain.

In order to estimate the dissipative energy of TA–PU hydrogels, loading–unloading cycle tests were conducted [47]. It can be seen from Figure 7A that the area of the hysteresis loop largened with the increase in the TA content. This suggested that these hydrogels effectively dissipated energy. It can also be observed from Figure 7B that the dissipative energy (U_hys_) of TA–PU hydrogels became greater with increase in concentration of the TA solution. When the concentration of TA solution increased up to 30 mg·mL^−1^, the dissipative energy of TA30–PU(S) hydrogel was almost 440 KJ·m^−3^, which was nearly seven times that of the pure PU hydrogel. The enhancement was due to the increase in crosslinking degree from numerous hydrogen bonds between TA and PU. For the pure PU hydrogel, the dissipative energy was predominated by its chemical crosslinking, namely originated from the deformation of crystalline domains and fracture of the chemical crosslinking points. However, the dissipated energy of TA–PU hydrogels was dominated by not only the first chemical crosslinking structure, but also the second physical crosslinking generated from hydrogen bonds.

### 3.7. Dynamic Mechanical Performance of TA–PU Gels

**Figure 8:**
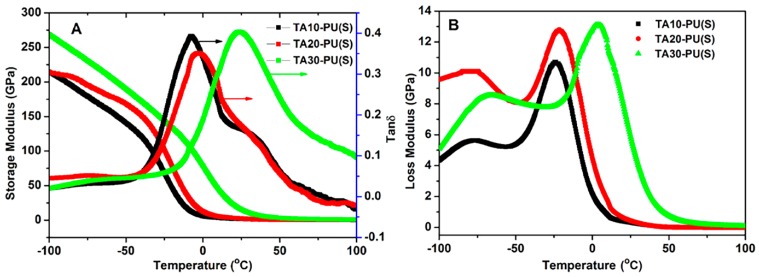
Temperature dependence of the storage modulus, tanδ (**A**) and loss modulus (**B**) of TA10(S)–PU, TA20(S)–PU, and TA30(S)–PU gels.

The mobility and viscoelastic performance of polymeric chains have a significant effect on mechanical property of hydrogels [48]. Therefore, we investigated the chain mobility and viscoelastic properties of TA–PU hydrogels by DMA. The traces of storage modulus (E′), loss modulus (E″) and loss tangent (tanδ) for TA10–PU(S), TA20–PU(S), TA30–PU(S) gels are shown in Figure 8. In Figure 8A, the E′s of all samples reduced as the temperature rose. As we noted, the E′ decrease rate of TA30–PU(S) gel was fastest, which illustrated that more hydrogen bonds led to easier movement of polyurethane chain segments because of the easy breakage and weaker entanglement of hydrogen bonds after heating; this benefited self-healing. Clearly, the TA30–PU(S) gel had a higher storage modulus than the other samples due to the crosslinking effect of more hydrogen bonds. Figure 8B shows the variety of E″ with the rise of temperature. It can be seen that the loss modulus of TA30–PU(S) gel was higher than that of the other gels owing to the increasing viscosity caused by the strong intermolecular interaction between TA and PU. Moreover, the glass transition temperatures (T_g_) acquired from corresponding tanδ peaks of TA10–PU(S), TA20–PU(S), TA30–PU(S) gels were −7.2, −2.4 and 24.23 °C, respectively (see Figure 8A). The T_g_s of these gels were increased with TA content, which again suggested that the increase of intermolecular interactions was caused by growth in amount of hydrogen bonds formed between TA and PU.

### 3.8. Self-Recovery and Self-Healing Properties of TA–PU Hydrogels

**Figure 9:**
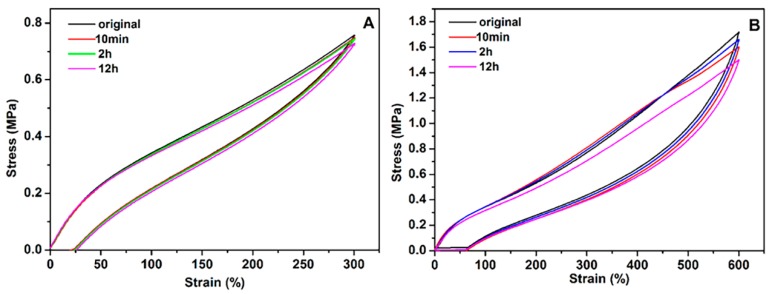
Tensile loading–unloading cycle curves of TA30–PU(S) hydrogel for different soaking times in distilled water: (**A**) at strain of 300%; (**B**) at strain of 600%.

The dynamic characteristics of hydrogen bonds can offer hydrogels favorable self-recovery ability. Hence, tensile loading–unloading cycle tests were conducted to examine self-recovery capability of TA–PU hydrogels. Figure 9A,B shows the loading–unloading results of TA30–PU(S) hydrogel at a strain of 300% and 600%. It can be seen from Figure 9A,B that the TA30–PU(S) hydrogel showed remarkable residual strain due to the structural damage in dissipative energy after the first loading–unloading cycle. However, the hydrogel nearly restored its initial properties when it was re-immersed in water. After immersing for 10 min, the second test was performed. For 300% of strain (Figure 9A), the loading–unloading loop was almost overlapped with the first one; while for 600% of strain, the loop recovery efficiency was 97%. After soaking for 12 h, the loop recovery efficiency of TA30–PU(S) hydrogel can restore to 96% at the 300% of strain, and 87% at the 600% of strain. As a result, the damaged inner network of TA–PU hydrogel can gradually self-recover. These observations indicated that the TA–PU hydrogels possessed excellent self-recovery capability.

The excellent self-recovery behavior of DC TA–PU hydrogels chiefly depended on the dynamic crosslinking caused by the hydrogen bonds between PU and TA. In the TA–PU hydrogels, the first crosslinking was constructed via a chemical crosslinker (TMP). The second crosslinking was formed by physical crosslinking (hydrogen bonding). When the small deformation occurred, the energy dissipation chiefly attributed to the fracture of hydrogen bonding, which can rapidly recover. However, when the large deformation occurred, the irreversible fracture of polyurethane chains would lead to only sectional self-recovery of the hydrogel.

The dynamic property of hydrogen bonds also imparted DC TA–PU hydrogels self-healing capacity. The TA30–PU(S) hydrogel was cut into two pieces, and the cut surfaces were pressed together. Subsequently, the gel was immersed into TA solution of 30 mg·mL^−1^ at 50 °C for 3 h. The tensile strength of self-healing hydrogel was 0.82 MPa with 400% of fracture strain (Figure S6). The healing efficiency of the gel was about 37%. Owing to plentiful hydrogen bonds existing between TA molecules and polyurethane chains, the fractured gel could heal spontaneously without external treatment.

### 3.9. Adhesiveness of TA–PU Hydrogels

**Figure 10:**
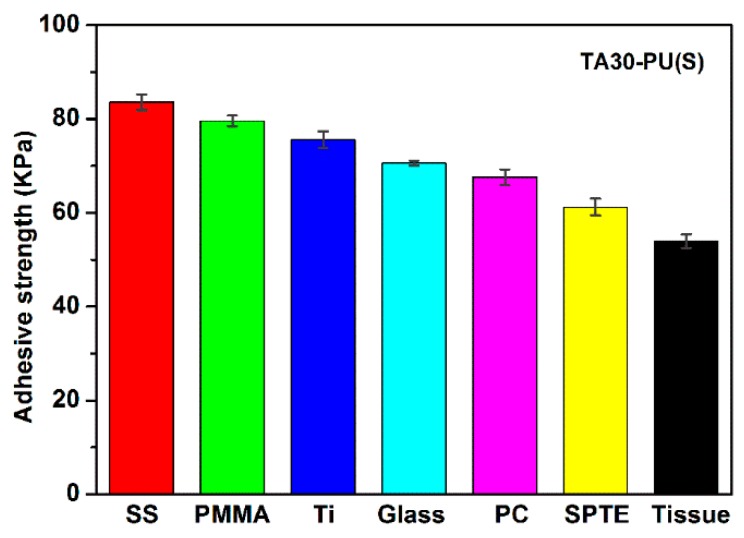
Adhesive strength of TA30-PU(S) hydrogels compared to stainless steel (SS), poly(methylmethacrylate) (PMMA), titanium (Ti), glass, polycarbonate (PC), tinplate (SPTE), and pork skin tissue.

At present, a great remaining challenge is to prepare a robust PU hydrogel with excellent adhesiveness [49]. Herein, we deliberately introduced TA into the PU hydrogel networks to increase their adhesiveness without compromising their mechanical properties, owing to abundant catechol groups present in the chemical structure of TA [35]. Catechol groups are capable of binding with the surfaces of organic and inorganic materials through forming irreversible covalent or reversible noncovalent interactions. As we can observe, while the adhesiveness tests were finished, the TA30-PU(S) hydrogel did not become fractured, but lost adhesion to the substrates due to the interfacial debonding. Consequently, the PU hydrogels with catechol groups possessed preferable adhesiveness [50,51]. The adhesive strength of TA30–PU(S) hydrogel to diverse substrates (metal, plastic, glass, animal tissue materials) are shown in Figure 10. The adhesive strengths were in a range of 54–85 KPa, indicating that the DC TA30–PU(S) hydrogel possessed adhesive capacity for various substrates.

## 4. Conclusions

A robust, tough and multifunctional DC PU/TA hydrogel was successfully fabricated by using a facile and versatile method. First, the initial network of PU was constructed through the chemical crosslinking of TMP. Second, TA was introduced into the PU network using a simple impregnation method to perform the second physical crosslinking via numerous hydrogen bonds, resulting in the formation of DC PU/TA hydrogels. In the DC TA–PU hydrogels, hydrogen bonding can dissipate more energy during breakage and contribute to the rapid reconstruction of network structure in times of PU hydrogel recovery and healing because it is a dynamic, invertible and sacrificial crosslinking process. The synergistic effect of double crosslinking led to the generation of double-crosslinked and single network PU hydrogels with high mechanical strength (2.3 MPa), outstanding toughness (9.6 KJ·m^−^^2^), favorable self-recovery and a self-healing ability. Furthermore, the tensile loading–unloading cycle test results indicated the self-recovery efficiency of PU30–TA(S) hydrogel reached up to 96% and 87% at the 300% and 600% of strain, respectively. The mechanism of stretched deformation of TA–PU hydrogels was further illustrated through utilizing the Mooney–Rivlin equation to analyze the stress–strain curves. Moreover, due to the special chemical character of TA, the TA–PU hydrogel possessed adhesiveness to various substrates and the adhesive strengths were in a range of 54–85 KPa. In summary, the novel DC PU/TA hydrogels with excellent mechanical properties and diverse functions were fabricated by a new means, which was practical, low-cost, and eco-friendly. As a result, the development PEG–PU hydrogels will benefit the biomedical field.

## Supplementary Materials

The following are available online at https://www.mdpi.com/2073-4360/12/1/239/s1, Figure S1: Variation of mass ratio with immersing time of porous PU hydrogels in different TA solutions. Error bars indicate standard deviation; N = 3. Figure S2: Variation of mass ratio with immersing time of porous TA10–PU hydrogel in distilled water. Error bars indicate standard deviation; N = 3. Figure S3: XRD graphs of PU and TAx–PU(S) dry gels. Figure S4: Swelling curves of PU(S) and TAx–PU(S) hydrogels. Figure S5: Images of adhesion test of TA30–PU(S) hydrogel. Figure S6: Tensile stress–strain curves of pristine and self-healed TA30–PU(S) hydrogels.
