# Supplementary material for: A Robust, Tough and Multifunctional Polyurethane/Tannic Acid Hydrogel Fabricated by Physical-Chemical Dual Crosslinking
Source: Polymers (Basel). 2020 Jan 19;12(1):239. doi: 10.3390/polym12010239 (PMC7023601; doi:10.3390/polym12010239)
Supplement: Supplementary file 1 [file polymers-12-00239-s001.pdf]

polymers

Supporting Information

# A Robust, Tough and Multifunctional Polyurethane/Tannic Acid Hydrogel by Physical-Chemical Dual Crosslinking

**Jie Wen [1], Xiaopeng Zhang [1], Mingwang Pan [1, 2,*], Jinfeng Yuan [1,2], Zhanyu Jia [1] and Lei Zhu [3]**

1 Institute of Polymer Science and Engineering, School of Chemical Engineering and Technology, Hebei University of Technology, Tianjin 300130, PR China; wenjie0329@126.com (J.W.); xpzhangup@163.com (X.Z.); yuanjf@hebut.edu.cn (J.Y.); 15122410536@163.com (Z.J.)
2 Hebei Key Laboratory of Functional Polymers, Hebei University of Technology, Tianjin 300130, PR China
3 Department of Macromolecular Science and Engineering, Case Western Reserve University, Cleveland, Ohio 44106-7202, United States; lxz121@case.edu
* Correspondence: mwpan@126.com

## 2. Experiment Section

### *2.1. Determination of the water content in TA-PU hydrogels*

The water content of as-prepared TA-PU hydrogels was calculated by the following formula:

$$WC = \frac{w_h - w_d}{w_h} \times 100\%$$

where $w_h$ is the mass of as-prepared TA-PU hydrogel, $w_d$ is the mass of corresponding dry gel.

The water content of TA-PU(S) hydrogels was obtained using the following formula:

$$WC = \frac{w_h - w_d}{w_h} \times 100\%$$

where $w_h$ is the mass of TA-PU(S) hydrogel, $w_d$ is the mass of corresponding dry gel.

### *2.2. Determination of the TA content in hydrogels*

The TA content of as-prepared TA-PU hydrogel was calculated by the following formula:

$$TC = \frac{w_{TA}}{w_h} \times 100\% = \frac{w - w_0}{w_h} \times 100\%$$

where $w_h$ is the mass of as-prepared PU-TAx hydrogel, w is the mass of corresponding dry gel. $w_0$ is the mass of porous dry gel before immersing in TA solution.

The TA content of PU-TAx(S) hydrogel was obtained using the following formula:

$$TC = \frac{w_{TA}}{w_h} \times 100\% = \frac{w - w_0}{w_h} \times 100\%$$

where $w_h$ is the mass of PU-TAx(S) hydrogel, w is the mass of corresponding dry gel. $w_0$ is the mass of porous dry gel before immersing in TA solution.

### *2.3. Healing percentage of PU-TAx(S) hydrogels*

Healing percentage of PU-TAx(S) hydrogels was obtained by the following formula:

$$\text{Healing Percentage (\%)} = \frac{\text{sample strength after healing (MPa)}}{\text{pristine sample strength (MPa)}} \times 100\%$$

### *2.4. Swelling ratio of PU-TAx(S) hydrogels*

$$S = \frac{m_s - m_d}{m_d} \times 100\%$$

where $m_d$ and $m_s$ are the mass of dry and swollen samples, respectively.

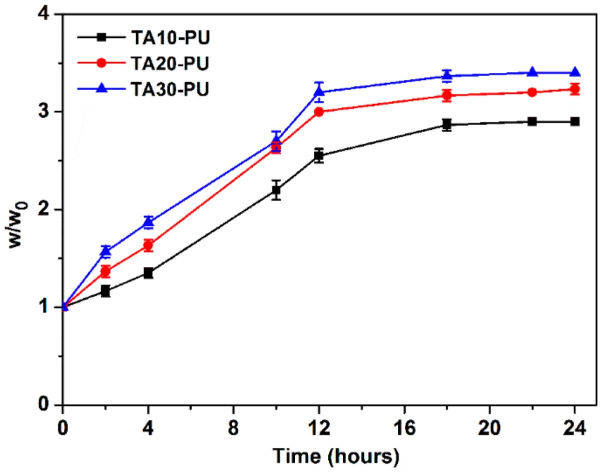


**Figure S1.** Variation of mass ratio with immersing time of porous PU hydrogels in different TA solutions. Error bars indicate standard deviation; N = 3.

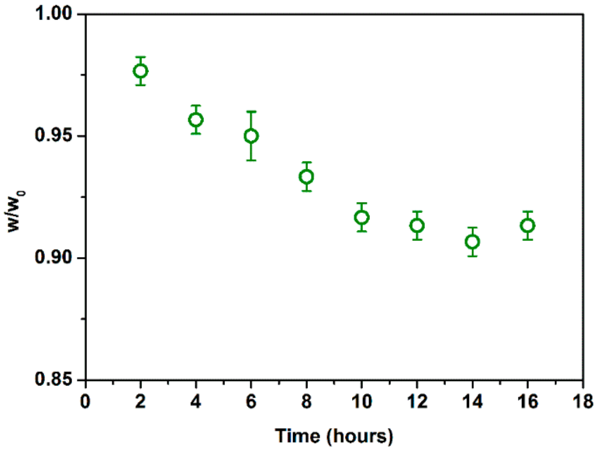


**Figure S2.** Variation of mass ratio with immersing time of porous TA10-PU hydrogel in distilled water. Error bars indicate standard deviation; N = 3.

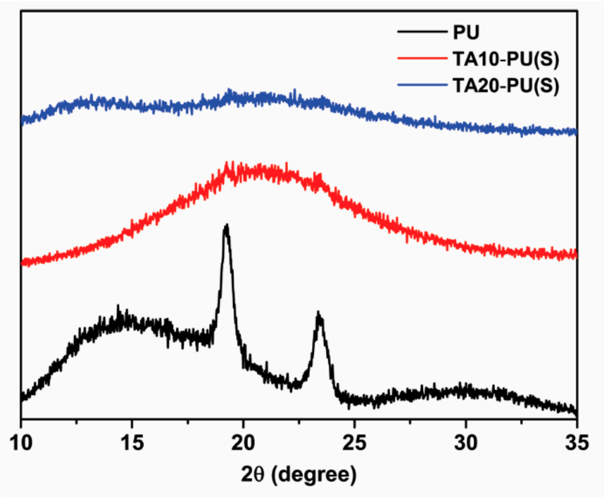


**Figure S3.** XRD graphs of PU and TAx-PU(S) dry gels.

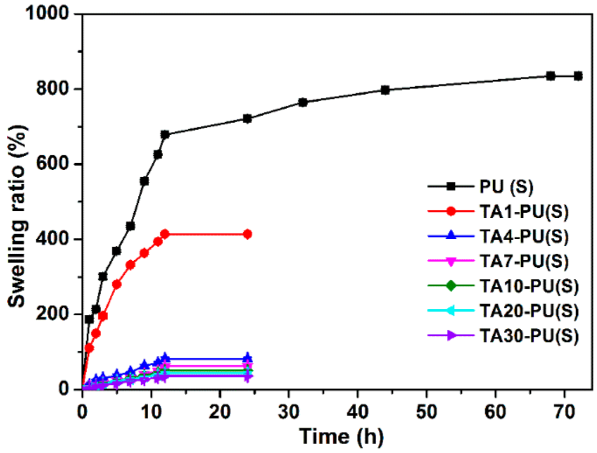


**Figure S4.** Swelling curves of PU(S) and TAx-PU(S) hydrogels.

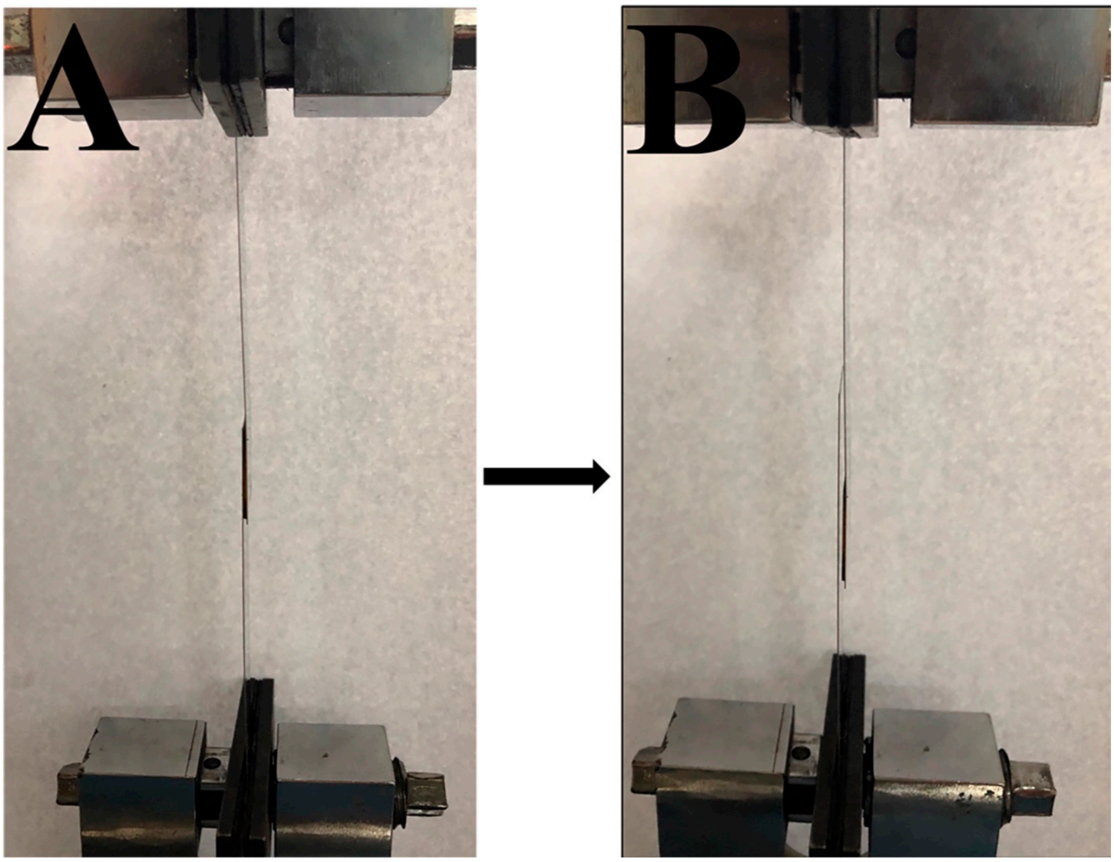


**Figure S5.** Images of adhesion test of TA30-PU(S) hydrogel.

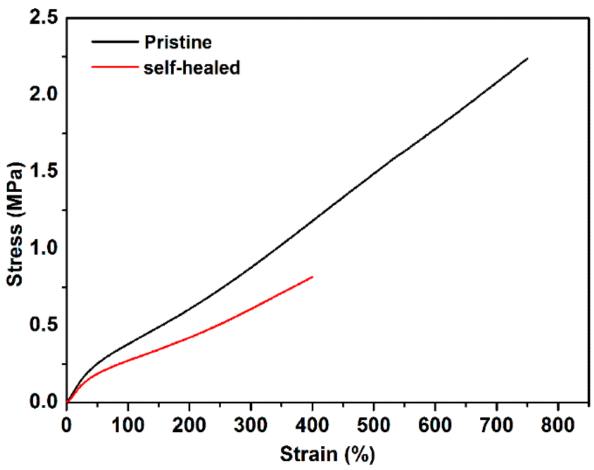


**Figure S6.** Tensile stress-strain curves of pristine and self-healed TA30-PU(S) hydrogels.

© 2020 by the authors. Licensee MDPI, Basel, Switzerland. This article is an open access article distributed under the terms and conditions of the Creative Commons Attribution (CC BY) license (http://creativecommons.org/licenses/by/4.0/).
